# A Star Network of Bipolar Memristive Devices Enables Sensing and Temporal Computing

**DOI:** 10.3390/s24020512

**Published:** 2024-01-14

**Authors:** Juan Riquelme, Ioannis Vourkas

**Affiliations:** Department of Electronic Engineering, Universidad Técnica Federico Santa Maria, Avda. España 1680, Valparaiso 2390123, Chile

**Keywords:** memristor, memristive device, star network, temporal memory, rank order code

## Abstract

Temporal (race) computing schemes rely on temporal memories, where information is represented with the timing of signal edges. Standard digital circuit techniques can be used to capture the relative timing characteristics of signal edges. However, the properties of emerging device technologies could be particularly exploited for more efficient circuit implementations. Specifically, the collective dynamics of networks of memristive devices could be leveraged to facilitate time-domain computations in emerging memristive memories. To this end, this work studies the star interconnect configuration of bipolar memristive devices. Through circuit simulations using a behavioral model of voltage-controlled bipolar memristive devices, we demonstrated the suitability of such circuits in two different contexts, namely sensing and “rank-order” coding. We particularly analyzed the conditions that the employed memristive devices should meet to guarantee the expected operation of the circuit and the possible effects of device variability in the storage and the reproduction of the information in arriving signal edges. The simulation results in LTSpice validate the correct operation and confirm the promising application prospects of such simple circuit structures, which, we show, natively exist in the crossbar geometry. Therefore, the star interconnect configuration could be considered for temporal computations inside resistive memory (ReRAM) arrays.

## 1. Introduction

Advances in the technology of resistive switching devices, also called “memristive” devices [1], are expected to bring innovation to the development of nonvolatile memory and relevant applications [2,3]. However, memristive devices are also considered an enabling technology for unconventional computing systems [4,5]. Among several such computing methods, in “race logic”, the information is represented with the timing of signal edges (i.e., wavefronts) instead of logic levels, and computation is performed by exploiting the delays between racing events. To the best of our knowledge, such a concept was introduced in [6], where a circuit design methodology was proposed for race computing, which demonstrated the best *area* × *delay* × *power* performance, compared to other conventional design approaches that rely entirely on binary logic and level-based logic computations. The race logic concept was revisited in [7] as a possible means to accelerate the solution to a broad class of optimization problems, by engineering race conditions in the circuits to perform computation. More recently, “space-time” algebra was proposed in [8], which captures the essential features of the race logic paradigm, providing a mathematical structure for modeling the relationships between events occurring in linear, discretized time, thus contributing to the design of race logic circuits, whose benefits for the solution of graph-based problems were discussed in [9]. Moreover, in [10], it was demonstrated that a functionally complete set of temporal operations can be realized in superconducting circuits, which can naturally compute directly over temporal relationships between pulse arrivals. Everything considered, the systematic exploration of this temporal computing scheme rests upon the development of “temporal memories”, which operate in the time domain based on wavefronts of signals and can store and reproduce temporally coded information. In this direction, a temporal memory cell design was presented in [11], which can linearly convert information from the time domain to a displacement on magnetic racetracks, using current pulses of varying lengths. The principles for wavefront propagation through metastable memristive transmission lines were presented earlier in [12,13], whereas a memristive crossbar array was used in [14] to store and recall wavefronts through tunable RC time constants. Even though the relative timing information of wavefronts can be captured via standard digital circuits before being stored in a memristive memory, as shown in [9,14], the rich collective dynamics of networks of memristive devices [15,16] could be leveraged for the development of memristive temporal memories to facilitate time-domain computations.

The dynamic response of memristive networks has been extensively explored for several potential applications [17,18]. Often, a specific configuration of bipolar memristive devices is used in much different contexts. For instance, two bipolar devices connected in series with opposite polarity (*anti-series*) could serve either as a memory cell [19,20] or as a voltage step sensor [21,22]. The suitability for each application depends on the performance characteristics that the employed memristive devices should satisfy. Moreover, it has been shown that complex interconnection patterns of memristive devices can be explored to achieve a conditional switching response [23].

In this context, here, we explore the star interconnect configuration of bipolar memristive devices for sensing and for arrival-time-coded computations, carried out in a memristive temporal memory fabric. Such a circuit exploits the rich analog dynamics arising in networks of memristive devices with different polarity. We particularly analyzed the conditions that the employed memristive devices should meet to guarantee the expected operation of the circuit and possible effects of variability in their switching performance. Depending on the switching characteristics of the individual memristive devices, the circuit could store the recent history of the arriving wavefronts in the resistance of the memristive devices and mark certain input channels before switching inhibition is activated. We simulated the proposed circuit configuration in LTSpice using a behavioral model of voltage-controlled bipolar memristive devices [24]. The simulation results validate the correct operation of the circuit and confirm the promising application prospects of such a simple device structure, which natively exists in the crossbar array geometry, so it is suitable for implementation in emerging resistive (memristive) memories.

## 2. The Memristive Star Network Topology

**Figure 1:**
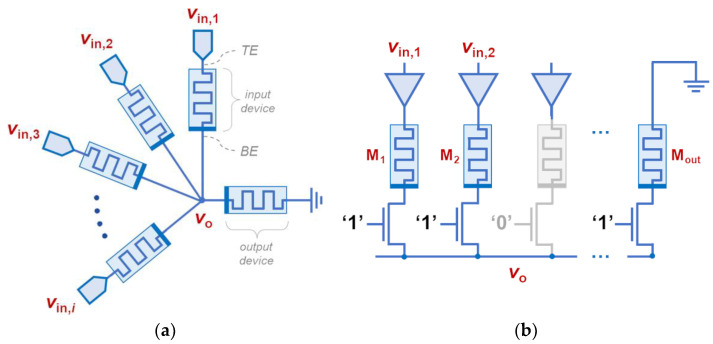
(**a**) Basic schematic of a star network of bipolar memristive devices. (**b**) Implementation of a star network within a row of a 1T1R crossbar array, by activating the select transistors of the memory cells and by properly driving the TEs. ‘1′ and ‘0′ represent the digital signals that are applied to the transistor gates to enable the corresponding branches of the star network.

An example of the proposed memristive star network topology is shown in Figure 1a. The circuit consists of *N* memristive devices whose top electrodes (TEs) are independent and their bottom electrodes (BEs) are commonly connected. We assume that when (V_TE_ − V_BE_) > V_SET_, the devices undergo a SET (resistance decrease) process, whereas a RESET (resistance increase) occurs when (V_BE_ − V_TE_) > |V_RESET_|. We consider *N* − 1 input terminals and only one output terminal. Thus, there are *N* − 1 memristive devices associated to the input channels and only one associated to the output terminal, which is connected to ground. During normal operation, the wavefronts of voltage signals arrive at the input terminals. The input memristive devices are originally in a high-resistive state (HRS) and can only undergo a SET process, whereas the output memristive device is initially in a low-resistive state (LRS) and can only experience a RESET process. A native formation of a star network within a 1T1R crossbar array is shown in Figure 1b to highlight its suitability for in-memory implementation.

The operation of this topology is based upon the dynamic response of a memristive voltage divider, as follows: When the first wavefront arrives at a specific input terminal IN*_i_*, a voltage divider is formed between the memristive devices M*_i_* and M_out_, which have opposite polarity. The larger portion of the applied input voltage *V*_in_ drops on M*_i,_* which is in HRS. Provided that (*V*_in*i*_ − *V*_o_) > V_SET_, it causes a SET process to M*_i_*, whose resistance drops to LRS. The resulting redistribution of the voltage between the two series devices triggers a RESET process for M_out_ once the voltage at the intermediate node *V*_o_ exceeds the |V_RESET_| threshold. From that moment on, if more wavefronts arrive, the M_out_ acts as a “fuse” since its HRS inhibits any change in the state of all the remaining input memristive devices. The reason is that the voltage *V*_o_ increases with the number of wavefronts that arrive, and this prevents the voltage drop (*V*_in*i*_ − *V*_o_) on the terminals of any input memristive device from reaching the value V_SET_.

## 3. Application-Specific Requirements for Memristive Devices

In an *anti-series* connection of memristive devices, the RESET is self-accelerating, since the higher the resistance of the device subjected to this process, the larger the voltage drop on its terminals. This leads to an increasingly faster switching rate towards HRS. On the contrary, the SET is self-limiting, since a decrease in the resistance of the device exposed to this process causes a proportional decrease to the voltage on its terminals. The latter can slow down the switching rate or even inhibit it. The target application, studied here, requires the RESET of M_out_ to be conditional to a previous SET of any input device. Therefore, the voltage threshold values and the HRS/LRS ratio of the employed devices should allow for this specific sequence of events. However, this also depends on the type of switching response (gradual or abrupt) of the devices during SET and RESET. For instance, since the purpose of M_out_ is to act as a “fuse”, its RESET process should be abrupt to suddenly interrupt any SET process of the input devices. Likewise, the type of SET response of the input devices may enable different applications for a memristive star network, as described below:

1. ***abrupt SET***: The arrival of the first wavefront will cause the conditional activation of the “fuse”. In the extreme case that many wavefronts arrive simultaneously, then the respective input memristive devices will all experience a SET process in parallel. Under these circumstances, the circuit can label the input channel(s) where the wavefront(s) arrived first, with the LRS of the respective memristive devices.
2. ***gradual SET***: The arrival of every successive wavefront will initiate a SET process in the corresponding input memristive device. If only one wavefront arrives, its SET process will eventually trigger the “fuse”. However, if more wavefronts arrive at close moments, several input memristive devices can be subjected to a SET process in parallel, before the “fuse” is activated. Under these circumstances, the circuit can capture the recent history of the arriving wavefronts and store such temporal information in the resistance of the input memristive devices using a “rank order” encoding scheme. Different resistive states will be achieved, proportional to the time that elapsed from the moment of arrival of the wavefront until the RESET of M_out_. The earlier a wavefront arrives, the lower the resistance of the corresponding memristive device(s).

The key difference in case (2) is that several input devices can change their state simultaneously until the RESET of M_out_ inhibits the switching process. However, the same is possible if the SET process is abrupt and the RESET process is gradual, as observed in the experimental measurements in [21]. In such a case, with the arrival of the first wavefront, the input memristive device will be abruptly SET to LRS and this will initiate the gradual RESET of M_out_ and the increase in the voltage at *V*_o_. Meanwhile, other arriving wavefronts could trigger the SET of the corresponding input memristive devices, as long as the resulting voltage drop on their terminals is higher than V_SET_. Thus, it becomes clear that the operation and the sensing possibilities of the proposed memristive star network topology depend on the type of response of the memristive devices and their overall performance.

Next, we identify the conditions that need to be satisfied for the memristive star network to operate as expected. It is necessary to find the maximum voltage amplitude allowed to be applied to the input terminals, which will guarantee that the state of the input memrsitive devices will not be modified after the “fuse” has been activated. To this end, we assume the worst-case scenario where all the memristive devices are in HRS, meaning that M_out_ has switched to HRS before any SET has previously occurred in the input memristive devices by the arriving wavefronts. For *k* identical input voltage sources of amplitude *V*_in_, it becomes *V*_o_ = *V*_in_ ∙ (*k*/(*k* + 1)). When only one wavefront has arrived (*k* = 1), the value of *V*_o_ is the lowest possible (*V*_in_/2), and there is a higher probability of potential drift induced to the resistance of the input memristive devices. This brings us to the maximum accepted amplitude for *V*_in_, which can be selected such that (*V*_in_/2) < V_SET_. Moreover, the HRS/LRS ratio of the devices affects the increment step of the resulting voltage on *V*_o_ with every arrival of a new wavefront. With a high resolution in the increment of *V*_o_, given a high |V_RESET_| threshold, many wavefronts need to arrive to create the conditions that will activate the memristive “fuse”. Thus, this is a circuit design parameter to consider.

## 4. Simulation Results

Here, we present results from circuit simulations, carried out using LTSpice, for different memristive device topologies. For the bipolar memristive devices, we used the behavioral model of voltage-controlled switching performance, proposed in [24]. If *V*_m_ is the voltage on the terminals of the memristive device and *V*_th_ is a voltage threshold, the resistance of the device is modified only if *V*_m_ > *V*_th_, and the switching rate is linearly dependent on the applied voltage, approximated by *β* × (*V*_m_ − *V*_th_), where *β* is a fitting constant. The values of the model parameters will be specified in each simulation scenario.

### 4.1. The Dynamic Memristive Voltage Divider

**Figure 2:**
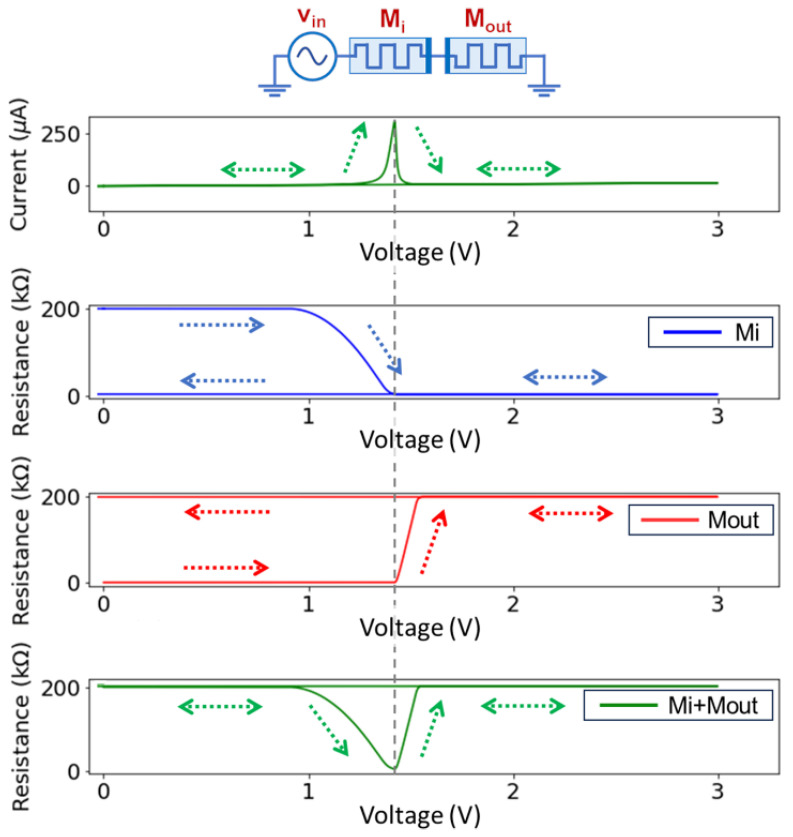
Simulation results for the performance of two *anti-series* memristive devices. The inset shows a schematic of the simulated circuit. The plots show the current through the two devices, the resistance of each one, and the sum of the two resistances, with respect to the applied voltage (*V*_in_). The arrows are a guide to the eye for the evolution of every measured parameter. The vertical dashed line indicates the moment when the completion of the SET of M_i_ triggers the RESET of M_out_.

The dynamic memristive voltage divider is the most fundamental structure within the star network topology. Moreover, the selected applications require the RESET of M_out_ to be conditional on a previous SET of an input memristive device. To this end, we first simulated the response of a voltage divider formed by the memristive devices M_i_ and M_out_, which have opposite polarity (see inset of Figure 2). The devices were properly initialized and then subjected to a positive triangular pulse (amplitude 3 V, rise time 100 ns). The values of the parameters of the model were selected as follows: R_ON_ = 1 kΩ, R_OFF_ = 200 kΩ, *β*_SET_ = *β*_RESET_ = 5∙10^13^ Ω/(V∙s), V_SET_ = 0.9 V, and V_RESET_ = −0.3 V. The simulation results are shown in Figure 2. Due to the high HRS/LRS ratio, almost all the applied voltage *V*_in_ initially drops on M_i_. Thus, as the applied voltage increases, the first device to receive sufficient voltage on its terminals to initiate its switching process is M_i_. We observe that the SET switching rate is slow at the beginning (self-limiting process). Once the SET of M_i_ is complete, only then are the conditions met for the RESET of M_out_ to be initiated. The RESET of M_out_ is faster than the previous SET of M_i_, because of its self-accelerated nature. The switching process is complete when *V*_in_ reaches approximately 1.5 V, and no further change is observed in the state of the two devices after that point. Therefore, we conclude that with the abovementioned device characteristics, the dynamic memristive voltage divider performs as expected. Note that similar circuit performance can be achieved with devices that have symmetric threshold values (V_SET_ = |V_RESET_|). However, if V_SET_ < |V_RESET_|, then the previous SET of any input memristive device is not the only condition for the RESET of M_out_ to start. In fact, after the SET of M_i_ is concluded, a higher input voltage is still required for the voltage drop across the terminals of M_out_ to exceed |V_RESET_|. So, higher amplitudes would be required to achieve the desired operation. Moreover, along with the threshold voltage values, the HRS/LRS ratio (namely R_OFF_/R_ON_ according to the naming of the model parameters) also affects the minimum voltage amplitude required to initiate the SET switching process of the input memristive device.

### 4.2. The Memristive Star Network

**Figure 3:**
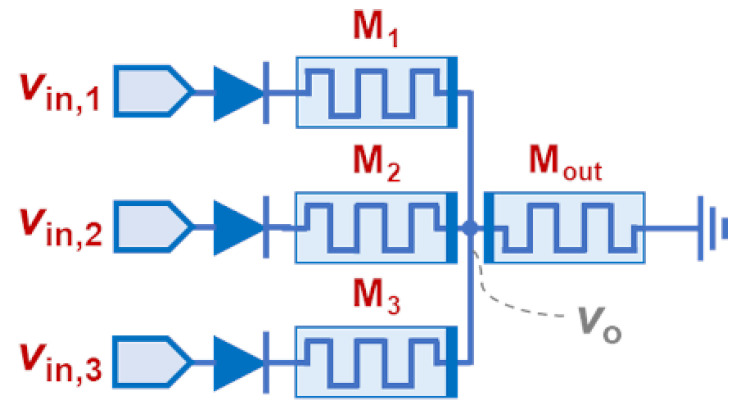
Schematic of the simulated memristive star network with three input channels.

Next, we focus on the performance of a memrsitive star network with three input channels. The simulated circuit is shown in Figure 3. In every input branch, in series with the input memristive device (M*_i_*), we used a diode to prevent the current flow towards the input terminals when the input voltage was at 0 V. The values of the memristive model parameters were kept as mentioned previously, except for the *β* parameters. Here, we used *β*_SET_ = 4∙10^10^ Ω/(V∙s) and *β*_RESET_ = 4∙10^12^ Ω/(V∙s), with a higher *β* for the RESET to comply with the memristive “fuse” concept, which requires the RESET to be as fast as possible. At the input terminals, we applied voltage pulses, which were 10-μs wide and had 2.2 V amplitude. Given a 0.7 V diode threshold, the effective voltage applied to the input channels was *V*_in_ = 1.5 V. Note that the V_SET_ = 0.9 V is higher than the effective *V*_in_/2, as required, whereas the value of V_RESET_ = −0.3 V guarantees that the RESET of M_out_ will not be triggered unless a complete SET process has first occurred in any input device. Note that such values of the model parameters reflect the performance features expected for any memristive device suitable to be used in such applications.

**Figure 4:**
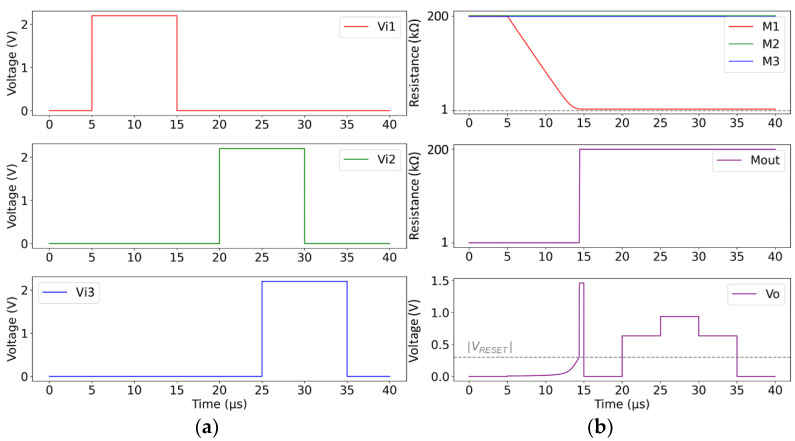
(**a**) Plot of the input voltage signals, applied to the three input channels of the star network. (**b**) Simulation results showing the time-evolution of the resistance of the input memristive devices (M_1_–M_3_), the output memristive device (M_out_), and of the voltage at node *V*_o_. The horizontal dashed line highlights the |V_RESET_| value.

In the first simulation scenario, we present the applied input signals in Figure 4a, whereas Figure 4b shows the evolution of the resistance of all the devices and the resulting voltage *V*_o_. Initially, only one input channel is activated. We observe that the corresponding device M_1_ switches gradually to LRS. As a result, the voltage *V*_o_ also increases, but there is a steep self-accelerated increase once *V*_o_ exceeds the RESET threshold of M_out_, which is attributed to the activation of the “fuse”. Next, when the second input channel is activated, the voltage on the respective memristive device is below its SET threshold because of the high *V*_o_ value. The same happens when two input channels are simultaneously active. This confirms the expected operation of the circuit. In fact, the input channel which first received a wavefront is correctly labeled, since M_1_ is the only input memristive device which eventually switched to LRS. Note that the number of inputs of the star network can be arbitrarily increased without any impact on the circuit operation.

**Figure 5:**
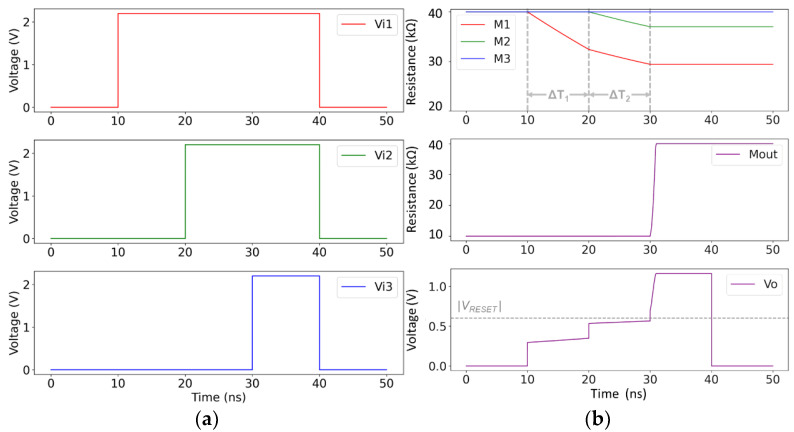
(**a**) Plot of the input voltage signals, applied to the three input channels of the star network. (**b**) Simulation results showing the time-evolution of the resistance of the input memristive devices (M_1_–M_3_), the output memristive device (M_out_), and of the voltage at node *V*_o_. The horizontal dashed line highlights the |V_RESET_| value. During ΔT_1_, only M_1_ is changing. During ΔT_2_, both M_1_ and M_2_ are changing their state, but at a much slower pace, compared to ΔT_1_.

In another simulation scenario, we explored the capability of “rank order” encoding of the temporal information of the arriving wavefronts. Regarding the memristive model parameters, here, we modified the *β* values (*β*_SET_ = 2∙10^12^ Ω/(V∙s), *β*_RESET_ = 1∙10^14^ Ω/(V∙s))) to achieve a switching time in the ns regime for the same amplitude of the input voltage signals. Moreover, the operational resistive range of all the memristive devices was reduced (R_ON_ = 10 kΩ, R_OFF_ = 40 kΩ) to evaluate the functionality of the circuit for a much lower HRS/LRS ratio. We also increased the |V_RESET_| threshold for representation purposes. We present the applied input signals in Figure 5a, and the resistance evolution of all the devices with the resulting voltage at node *V*_o_ in Figure 5b. The arriving wavefronts are purposely spaced 10 ns apart. The arrival of every wavefront initiates a SET process only to the memristive device of the corresponding input channel. After 30 ns, the arrival of the last wavefront (V*_i_*_3_) instantly triggers the “fuse”, so the device M_3_ is unable to significantly modify its resistance. It can be noted that the circuit is able to achieve a “rank order” encoding, since the order of arrival of the wavefronts is correctly captured. The earlier the wavefront arrives, the lower the resistance of the corresponding memristive device.

When a “timing code” scheme is required, then equally spaced wavefronts should be represented by equally spaced resistive values in the state of the input memristive devices. Nevertheless, with a closer observation of Figure 5b, we conclude that a “timing code” is not possible to achieve with this memristive star network topology. In the memristive model we used [24], the change rate of the resistance depends linearly on the voltage across the device terminals, i.e., *V*_m_ = *V*_in*i*_ − *V*_o_ for the input memristive devices. So, if the voltage at *V*_o_ increases, *V*_m_ decreases and the switching rate of the input memristive devices is slowed down. Indeed, we notice in Figure 5b that every arrival of a new wavefront causes a sudden increase in the voltage at *V*_o_, which leads to a drastic decrease in the rate of change in all the devices that are subjected to a SET process (we observe a “break” in the curves of M_1_ and M_2_ with a notably different slope during ΔT_1_ and ΔT_2_). Therefore, the order of arrival is the only information that can be stored in the input memristive devices of a memristive star network topology as the one proposed in this paper.

### 4.3. A Circuit for Capture and Reproduction of Signal Edges

**Figure 6:**
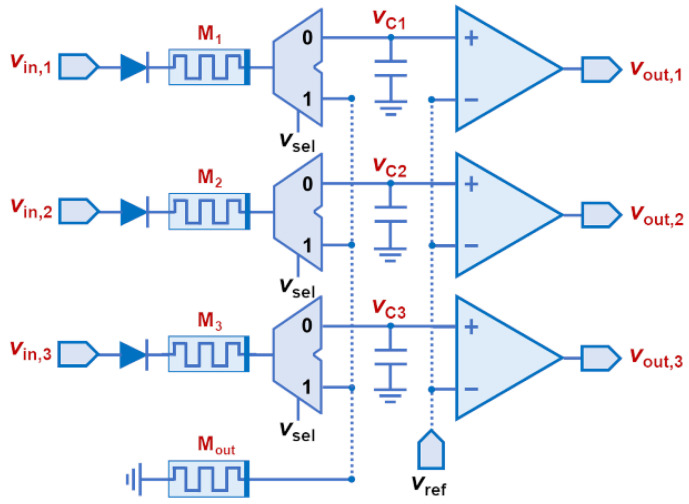
Schematic of the simulated circuit, able to capture and reproduce voltage wavefronts.

**Figure 7:**
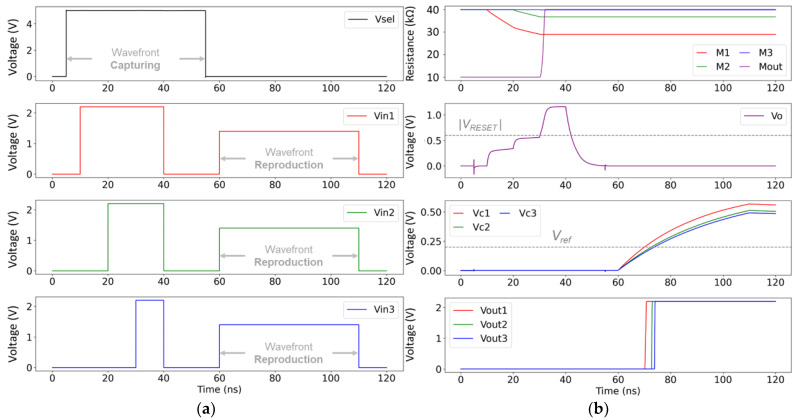
(**a**) Plot of the input voltage signals, applied to the three input channels, and the selection signal V_sel_ of the MUXes. (**b**) Simulation results showing the time-evolution of the resistance of all the memristive devices, the voltage at node *V*_o_, and the voltage at the input/output nodes of the comparators. The comparison threshold V_ref_ is shown with a dashed line.

In the last simulation scenario, we demonstrate both the capture and the reproduction of wavefronts by properly driving the memristive star network. The simulated circuit is shown in Figure 6. Compared to the previous design in Figure 3, here, we included a set of custom analog multiplexers (MUXes) designed with 1 μm CMOS transistor models to selectively connect the memristive devices that hold the wavefront information to the analog comparators of the output stage. For the comparator modules, we used a behavioral description in LTSpice. We show in Figure 7a the applied voltage signals and, in Figure 7b, the simulation results, including the input/output voltage of the comparator modules. The memristive model parameters were kept as in the previous simulation scenario. The parasitic capacitance of the memristive devices was not taken into account. However, the parasitic capacitance of the transistors, used to implement the analog MUXes was properly considered in simulations, whereas the capacitors shown in the circuit of Figure 6 aim to represent the total capacitance of the output lines.

When V_sel_ is ‘1’, the MUXes allow the memristive star network to be formed, so the arrival of wavefronts can be stored in the memristive devices. Unlike in Figure 5b, we note that the parasitic capacitance of the transistors, implementing the MUXes, produces a different shape in the response at node *V*_o_. However, the switching performance of the memristive devices is very similar to what was observed and discussed in previous simulations, so the information of the arriving wavefronts is correctly stored. Next, when V_sel_ is ‘0’, the input channels are connected to the network of comparators at the output stage. This circuit is based on a previous proposal in [14]. When the same voltage pulse is applied to all the input channels, the capacitors of the output lines are charged via the memristive devices. Different resistance values in the memristive devices generate different *RC* time constants, so the moment the comparison threshold V_ref_ is reached is different in every comparator and depends on the previously captured timing characteristics of the incoming wavefronts. The latter produces a varied response at the output nodes V_out1_ through V_out3_ that allows for reproducing the order of the previously received wavefronts, as we can observe in Figure 7b. Note that the order of arrival is the only information that can be encoded in the state of the input memristive devices. The only way to achieve matching of the capturing and the reproduction time scales is by using variable capacitor values at the output lines. This, however, is not further explored in this work.

Even though the precise timing characteristics of the input waveforms cannot be recovered from the information stored in the memristive devices, the simulation results confirm the correct operation of the proposed circuit, representing a solution for sensing and for arrival-time-coded computations, able to be carried out inside the core of a resistive memory. In this context, note that the proposed application does not imply any significant area overhead, since the MUX and the comparator modules can be found in the periphery of a resistive crossbar array, as part of the driving circuitry used to perform conventional READ and WRITE memory operations (see [5] for further information of such a driver).

### 4.4. Effects of Memristive Device Variability in the Overall Circuit Performance

Variability in the switching performance is an important aspect of memristive devices, as analyzed in [25]. Therefore, to test the correctness of the circuit response in the presence of variability, we upgraded the model of memristive devices to incorporate *cycle-to-cycle* and *device-to-device* variability. More specifically, up to 50% variability was considered for the threshold parameters (V_SET_ and V_RESET_) and for parameter *β*.

Note that, for the circuit to be able to label the input channel(s) where the wavefront(s) arrived first and to capture the recent history of the arriving wavefronts, the RESET of the output device should be conditional on a previous SET of an input device. To this end, the relation of the switching thresholds of the employed devices is very important. If the variability makes V_SET_ < |V_RESET_|, then the previous SET of any input memristive device will not be the only condition for the RESET of the output device to start. To avoid such a situation, the most appropriate devices to consider for this implementation should demonstrate a V_SET_ threshold sufficiently higher than |V_RESET_|. Moreover, it was explained that the amplitude for the *V*_in_ pulses can be selected such that (*V*_in_/2) < V_SET_. If such a relation does not hold, owing to variability, then it cannot be guaranteed that the state of the input memrsitive devices will not be modified after the “fuse” has been activated. Therefore, the selected *V*_in_ amplitude in the abovementioned relation should consider the percentage of observed variability in the V_SET_ threshold of the input devices. Everything considered, the variability in the switching thresholds can principally affect the “fuse” function, since the output device could be activated earlier than expected and prevent the input devices from switching sufficiently their state, or the input devices could keep changing their state even after the RESET of the output device has occurred.

Furthermore, regarding the “rank order” encoding, the earlier a wavefront arrives at the input channel, the lower the achieved resistance of the corresponding input memristive device. However, variability applied to the fitting constant *β* directly impacts on the switching rate of the devices and could possibly affect the “rank order” encoding capacity of the circuit. More specifically, memristive devices that received the wavefront later could unexpectedly achieve a lower final resistance. Consequently, the temporal information of the arriving wavefronts could be stored in the wrong order. Nevertheless, our analysis showed that this is only possible for arriving wavefronts that are too close to each other. Everything considered, the minimum distinguishable spacing of the arriving wavefronts emerged as another circuit design parameter to consider, owing to variability.

## 5. Conclusions

Through circuit simulations, we validated the application prospects of the rich analog dynamics arising in a star network of bipolar memristive devices. We particularly demonstrated the suitability of the proposed circuit in two different contexts, namely for *sensing* and “*rank-order*” *coding*, even though the relationship between the input signal duration and the corresponding resistance of the storage elements is not linear. Other works previously used standard digital circuits to capture the relative timing information of arriving wavefronts. Alternatively, the circuit proposed in this work could directly store the recent history of the arriving wavefronts in the resistance of memristive devices. Possible limitations for the target application include the device variability and the nonlinear time evolution of the resistance of the devices under constant voltage biasing. The latter could impact on the resolution of the system for the storage and the reproduction of the arriving signal wavefronts, so they should be taken into consideration along with the rest of the design parameters, commented and analyzed throughout this work, for the design of sensing and temporal memory structures based on memristive star networks. Of course, the driver capacitance, the wire resistance and capacitance, as well as post-layout design information and technology-specific models of memristive devices should be included in any detailed analysis concerning the scaling of such temporal circuits. Future work includes the further exploitation of possibilities of memristive star networks in hardware with real memristive devices.

## Data Availability

No data available online. The data that support the findings of this study can be available from the corresponding author upon reasonable request.
